# Systematic Optimization of the Synthesis of Confined Carbyne

**DOI:** 10.1002/smtd.202500075

**Published:** 2025-04-09

**Authors:** Clara Freytag, Christin Schuster, Emil Parth, Dido Denier van der Gon, Takeshi Saito, Kazuhiro Yanagi, Paola Ayala, Thomas Pichler

**Affiliations:** ^1^ University of Vienna Faculty of Physics Boltzmanngasse 5 Vienna 1090 Austria; ^2^ National Institute of Advanced Industrial Science and Technology (AIST) Nanomaterials Research Institute 1‐1‐1 Higashi Tsukuba Ibaraki 305‐8565 Japan; ^3^ Tokyo Metropolitan University Department of Physics 1‐1 Minami‐Osawa Hachioji Tokyo 192‐0397 Japan

**Keywords:** carbyne, confined carbyne, carbon nanotubes, Raman spectroscopy, synthesis

## Abstract

Confined carbyne, an sp^1^‐hybridized linear carbon chain inside a carbon nanotube, is a novel material with remarkable properties and potential applications. Among its currently successful synthesis methods, high temperature high vacuum annealing is prevalent. Further optimization could be achieved by tuning the synthesis pathway. Here, a systematic analysis of key synthesis parameters including precursor filling, annealing step sequences, and temperature conditions during high temperature vacuum processing is performed. A novel yield determination model that overcomes previous limitations related to the irregular resonance Raman behavior of carbyne is applied to evaluate bulk yield and realized growth potential. With this refined model, it is possible to make a quantitative assessment of bulk yield optimization potential in multi‐step annealing processes. These results provide crucial insights into the fundamental formation mechanisms of confined carbyne, advancing our understanding of this promising hybrid nanomaterial system. It is therefore possible to establish improved protocols for maximizing confined carbyne yields through precise control of synthesis conditions.

## Graphical abstract

The bulk yield of confined carbyne inside carbon nanotube hosts is governed by the synthesis parameters such as temperature, pressure, filling with carbonaceous precursors and number of oxidation and annealing steps. This work quantifies the influence of these parameters using a recently developed model for yield determination and elucidates the mechanisms governing the growth of confined carbyne.

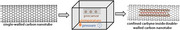

## 1 Introduction

Carbyne is a nanomaterial consisting of an infinite chain of linear sp^−1^‐hybridized carbon atoms. As a true 1D material, it has been predicted to have remarkable optical, electronic and mechanical properties.^[^ ^1^, ^2^, ^3^, ^4^, ^5^, ^6^, ^7^, ^8^ ^]^ The linear carbon chains can either have continuous double bonds or alternating single‐triple bonds with different lengths, which is more stable due to Peierl's distortion.^[^ ^9^ ^]^ The bond length alternation (BLA), i.e., the difference between the length of the single and triple bond, influences the properties of the carbon chains, such as the bandgap.^[^ ^10^ ^]^ While the cumulenic structure (double bonds, BLA = 0) is metallic, the polyynic structure (single and triple bonds, BLA ≠ 0) is a semiconductor with a tunable band gap.^[^ ^11^ ^]^ Many properties of linear carbon chains are length dependent. The term carbyne refers to a material with an “infinitely” long chain, i.e., where the properties become independent of length.^[^ ^12^ ^]^ The main issue with this material is the lack of stability due to the exothermic collapse of the chain or cross‐linking with other chains.^[^ ^13^ ^]^ End‐capping groups can prevent dangling bonds and sterically hinder the collapse of a chain onto itself while spacers impede reaction of chains with each other.

There are many different approaches to synthesize and stabilize linear carbon chains. Polyynes with around 20 carbon atoms end‐capped by hydrogen or nitrogen were synthesized by laser vaporization of graphite,^[^ ^14^ ^]^ in solution using laser ablation of graphite^[^ ^15^, ^16^ ^]^ and by arc‐discharge between graphite electrodes.^[^ ^17^ ^]^ Organic “bottom‐up” synthesis methods have yielded cumulenes with up to 10 carbon atoms^[^ ^12^ ^]^ and polyynes with up to 44 carbon atoms^[^ ^18^, ^19^ ^]^ using large organic end‐capping groups for stabilization. Recently, with additional stabilization of the chain during synthesis using masking groups and threading the chains through organic macrocycles, even longer chains with up to 68 carbon atoms have been synthesized using this pathway.^[^ ^20^ ^]^

Atomic manipulation methods can also lead to interesting structures containing linear carbon. In a transmission electron microscope (TEM), carbon atoms can be removed from a graphene sheet until only a chain remains between two patches of graphene.^[^ ^21^, ^22^ ^]^ Carbon chains can also be created in situ from graphene flakes by mechanically pulling them apart after electrical contacting to a gold tip inside a TEM.^[^ ^23^ ^]^ Inside a field ion microscope (FIM), high voltage can be applied to a needle‐shaped sample of carbon‐based fibers, unraveling chains of carbon from the tip.^[^ ^24^ ^]^ Recently, a chain with 120 carbon atoms was manufactured in situ using an atomic force microscope (AFM) by demetallization of an organometallic polyyne precursor on a gold surface.^[^ ^25^ ^]^

A first pathway to carbyne was found by stabilizing the carbon chain inside small‐diameter double‐walled carbon nanotubes (DWCNTs).^[^ ^26^ ^]^ The interaction of the carbyne chain with the host tube stabilizes the chain and sterically prevents the cross‐reactions of the chain with itself. In recent years, different synthesis routes for confined carbyne inside carbon nanotubes (CC@CNT) have been developed.^[^ ^27^, ^28^, ^29^, ^30^ ^]^ The most common methods are based on high temperature annealing in high vacuum (HV). Confined carbyne (CC) can grow directly inside as‐grown chemical vapor deposition (CVD) DWCNTs^[^ ^26^ ^]^ or enhanced direct injection pyrolitic synthesis (eDIPS) single‐walled carbon nanotubes (SWCNTs).^[^ ^31^ ^]^ In the case of direct annealing of SWCNT hosts, the inner walls are formed during the annealing, together with the carbyne. The growth of carbyne inside the host CNTs is limited by the availability of carbonaceous precursor. Some amount of carbon feedstock is generally present inside the CNTs from their synthesis, but more can be supplied by filling the host tubes with small organic molecules such as methanol,^[^ ^30^ ^]^ fullerenes^[^ ^29^ ^]^ or short chain linear polyynes^[^ ^32^, ^33^, ^34^ ^]^ or by oxidizing the DWCNT host to create defects.^[^ ^35^ ^]^ Photothermal synthesis is an alternative to high temperature annealing in HV.^[^ ^28^ ^]^ Recently, low temperature annealing has been used to synthesize weakly confined carbyne from surfactant molecules^[^ ^36^ ^]^ or aromatic hydrocarbons^[^ ^37^ ^]^ as carbonaceous precursors inside SWCNTs.

In this work, we focus on the quantification of the influence of different parameters in the synthesis of CC. The growth of CC is governed by three main factors: a) availability of carbonaceous precursors inside the host nanotube, b) annealing temperature, and c) residual gas pressure in the furnace. Additionally, the achievable bulk yield is limited by the fraction of carbon nanotube hosts with the appropriate diameter among the diameter distribution of the specific sample.^[^ ^38^ ^]^ Therefore, it is possible to evaluate the realized growth potential achieved by relating the bulk yield to the fraction of fillable tubes, providing a useful metric to evaluate the success of the synthesis process.

## 2 Results and Discussion

### 2.1 Influence of Providing Additional Carbonaceous Precursor via Filling

The growth of confined carbyne is partially governed by the availability of carbonaceous feedstock in the host nanotubes. This led us to evaluate the effect of filling carbonaceous precursors inside small diameter SWCNTs. As an exemplary case study, the CC bulk yield after annealing of unfilled and C_60_ filled 1.45 nm arc‐discharge SWCNTs was compared. For filling, the CNTs were first oxidized in air at 450°C for 30 min to open the end‐caps. Next, the sample and the fullerenes were placed in a glass ampoule which was then evacuated to HV and placed inside a furnace for 12 h at 550°C to create peapods. Both the peapods and the unfilled sample were placed into a ceramic furnace together for annealing to ensure the same experimental conditions during the annealing step.

**Figure 1:**
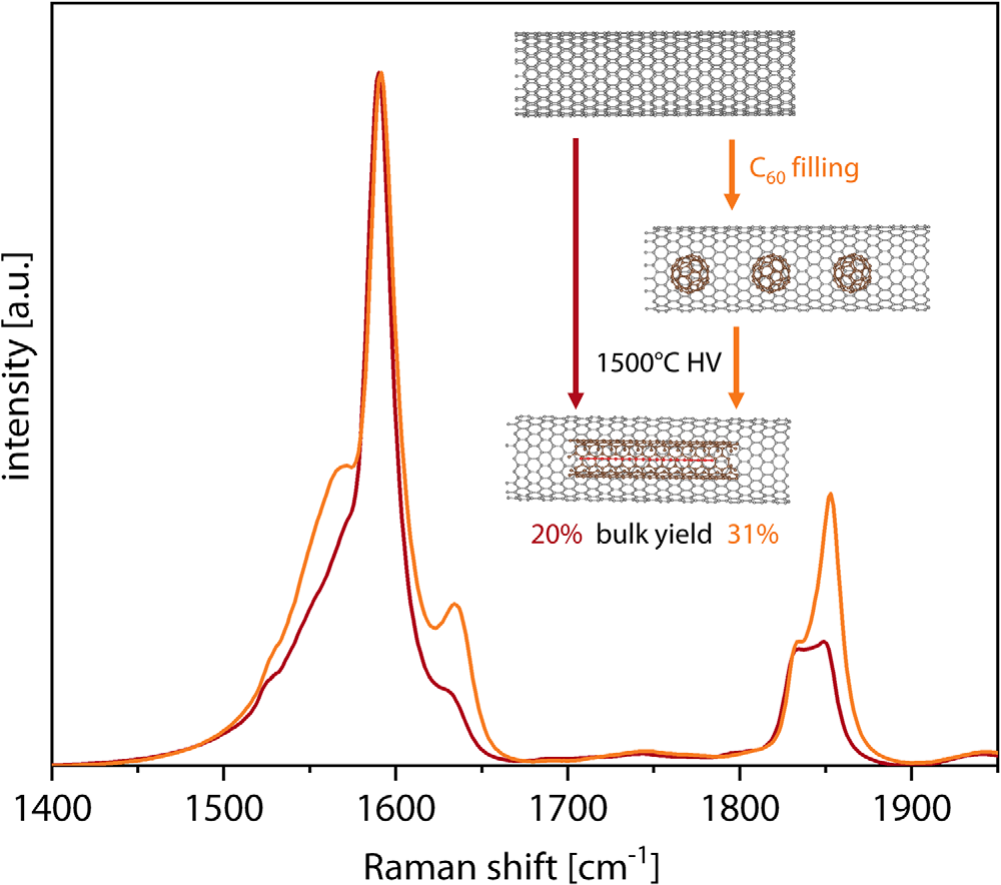
Effects of filling carbon nanotube hosts (1.45 nm arc‐discharge) with C_60_ on CC yield: Raman spectra of CC from an unfilled nanotube host (red) and from peapods (orange), measured with a 568 nm laser. The bulk yield increases from 20% to 31%. A schematic of the synthesis process for the filled versus unfilled host tubes is also shown.

In **Figure** **1**, Raman spectra of CC@DWCNTs grown from the unfilled and the C_60_ filled hosts are shown. In the Raman spectra, changes in the peak attributed to the CC at around 1850 cm^−1^ and to the G‐line of the host DWCNT can be observed. The CC‐peak is made up of multiple components according to the geometry of the surrounding inner host tube.^[^ ^39^ ^]^ Depending on the growth rate inside the different inner tubes, combined with the narrow resonance window and strong resonance enhancement of the CC‐mode, the components may appear with different intensities. This demonstrates the unreliability of using the CC‐mode for yield determination. The growing left and right shoulders in the G‐lines were recently explained by anharmonic phonon‐phonon interaction as coupled modes between the host nanotube and the carbyne chain.^[^ ^40^ ^]^ While the CC‐peak is strongly resonance dependent, the G‐line shoulders follow the more stable resonance behavior of the chain and can therefore be reliably used to determine the bulk yield. The coupled mode evaluation model is described in detail in Section 4 and the work of Schuster *et al.* ^[^ ^38^ ^]^ Using this method, an improvement in the bulk yield from 20% to 31% was determined when filling the SWCNT hosts with C_60_ as a carbonaceous feedstock.

### 2.2 Improving the Yield via a Multi‐Step Annealing Process

Two‐step annealing processes have previously shown to improve the CC yield.^[^ ^29^, ^35^ ^]^ To quantify the influence of a second annealing step and the introduction of a double‐walled intermediate, peapods made from 1.45 nm arc‐discharge CNTs were used as hosts for two different synthesis pathways. One half of the original sample was annealed in HV for 1 h at 1500°C to immediately grow CC. The other half was annealed in HV for 2 h at 1300°C in order to first only grow DWCNTs as an intermediate. In both cases, the maximum length of CNTs where inner walls (and CC) can be grown is around two thirds, governed by the amount of fullerenes inside. In the following step, both halves were placed in a furnace and oxidized at 500°C for 1 h at 600 mbar of air, where the single‐walled parts are preferentially destroyed, as SWCNTs are less stable than DWCNTs at this temperature.^[^ ^41^ ^]^ The free carbon from etching can be used to form inner walls and CC in the remaining parts of the tubes, which further increases the fraction of DWCNTs in the samples. The oxidation step also ensures opening of the end‐caps of the inner tubes and partial destruction of the DWCNTs and introduces defects which help the growth of carbyne.^[^ ^35^ ^]^ While the oxidation step leads to a fracturing of long nanotubes, the resulting segments are still of sufficient length for hosting carbyne. In the second annealing step, both halves of the sample were annealed at 1500°C for 1 hour in HV to grow carbyne.

**Figure 2:**
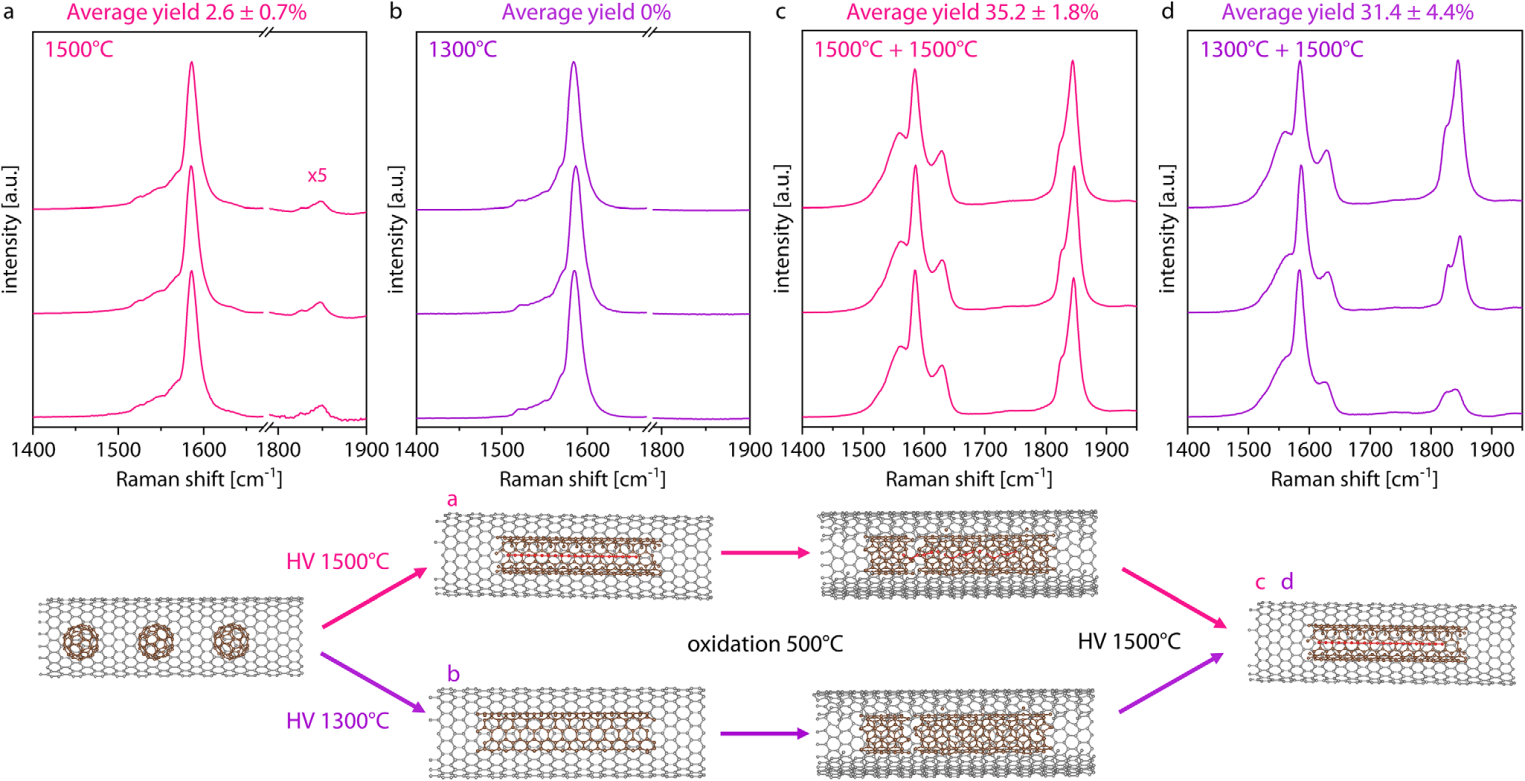
Two‐step synthesis process for CC grown from 1.45 nm arc‐discharge peapods: Raman spectra of 3 spots on the sample after a) annealing once at 1500°C; b) annealing once at 1300°C; c) annealing twice at 1500° C with an oxidation step in‐between; d) annealing first at 1300° C and then at 1500° C with an oxidation step in‐between. All Raman spectra were measured with a 568 nm laser. Below, the process of carbyne growth from peapods is shown schematically.

Raman spectra were taken after the first and second annealing step for both pathways (**Figure** **2**). After the first annealing step, there is only a very small peak visible for CC (Figure 2a) and the G‐line very closely resembles the G‐line area of the DWCNTs made by annealing once at 1300°C (Figure 2b). After the second annealing step, both samples showed strong CC peaks and the characteristic G‐line shoulders resulting from coupled modes appear for both samples. The evaluation of the G‐line area and subsequent calculation of bulk yield showed that both samples have a similar average bulk yield of 35% for the 1500°C+1500°C pathway and 31% for the 1300°C+1500°C pathway. The fraction of theoretically fillable host nanotubes of the 1.45 nm arc‐discharge CNTs was calculated to be 48% from optical absorbtion data (see Supporting Information S3). The realized growth potential, relating the bulk yield to this maximum fillabilty of the host nanotubes, was determined to be 73% and 65% for the two pathways, respectively, with the two‐step method of annealing from peapods.

### 2.3 Analysis of Multiple Annealing Steps: How Close Can We Get to The Maximum Fillability

**Figure 3:**
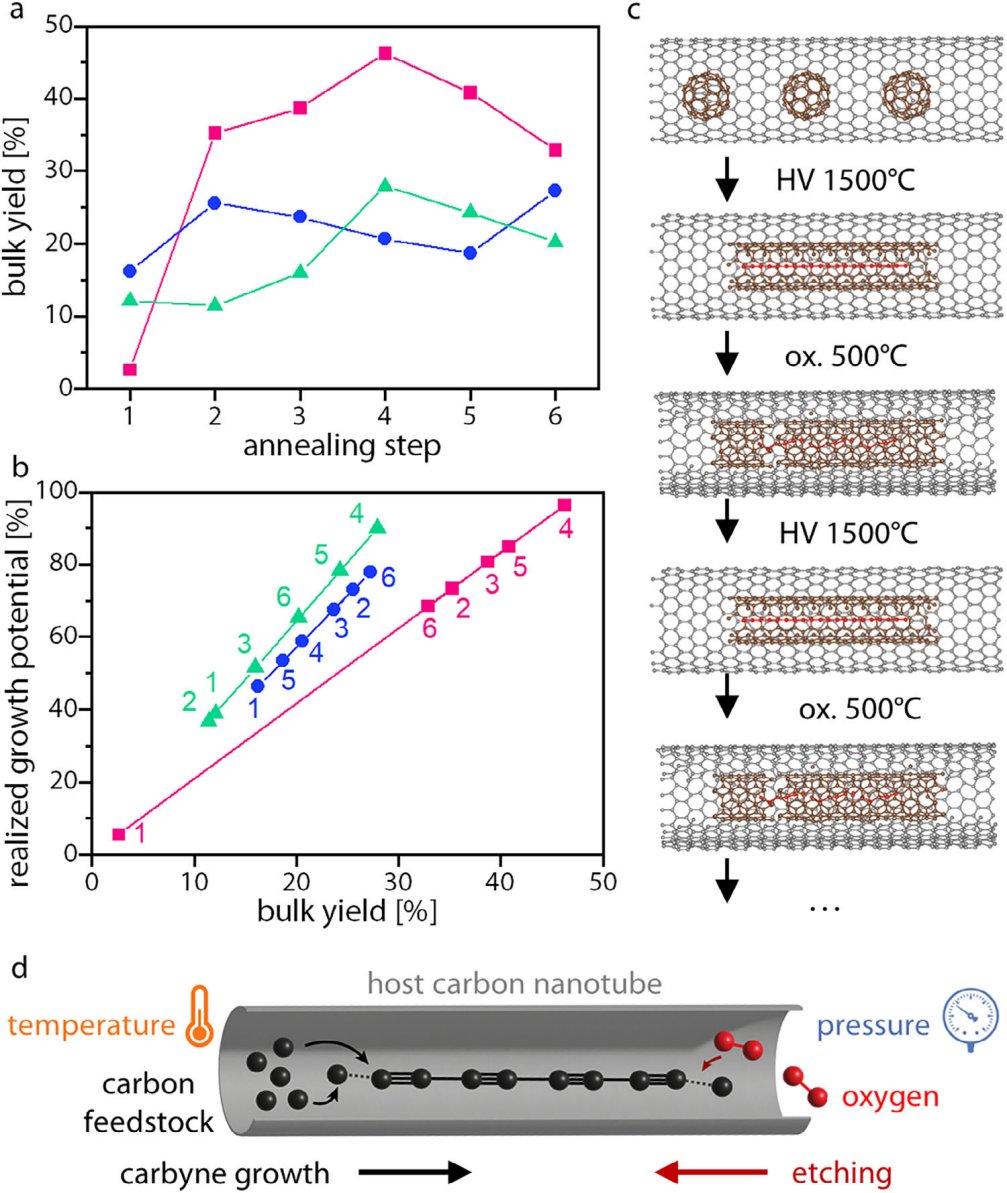
Multi‐step synthesis of CC inside different CNT hosts: a) bulk yield of CC inside 1.45 nm arc‐discharge (pink square), 1.33 nm eDIPS (green triangle) and 1.41 nm eDIPS (blue circle); b) realized growth potential for the different CNT hosts; c) schematic of the multi‐step annealing process from peapods; d) growth model for CC: two processes compete with each other ‐ formation of the carbyne chain, which is governed by the temperature and availability of carbonaceous feedstock, and etching/destruction of the carbyne chain due to residual gas in the furnace (i.e., pressure).

Since it was shown that two annealing steps with oxidation in‐between can improve the bulk yield of CC, it was tested if more repetitions will further improve the yield. This experiment was conducted with three types of CNT hosts: 1.45 nm arc‐discharge nanotubes, 1.33 nm eDIPS nanotubes and 1.41 nm eDIPS nanotubes. The 1.45 nm arc‐discharge and 1.41 nm eDIPs nanotubes were filled with C_60_ fullerenes prior to the first annealing step. All samples were then annealed a total of six times in HV for 1 hour at 1500° C, with an oxidation step at 500° C for 1 h at 600 mbar of air between annealing. Raman spectra were taken after each annealing step for each of the CNT hosts (**Figure** **3**).

For the 1.45 nm arc‐discharge and the 1.33 nm eDIPS CNT hosts, the highest bulk yield was achieved after four annealing steps, with 46% and 28%, respectively (Figure 3a). Further annealing did not lead to a higher bulk yield as during annealing, two processes compete with each other. On one hand, carbyne is formed from carbonaceous precursors inside the DWCNTs, increasing the bulk yield. On the other hand, the residual gas inside the furnace destroys the inner nanotubes and the carbyne chains, leading to a decrease in the bulk yield (Figure 3d). Whether the yield will increase or decrease by further annealing depends on the rate at which the two processes occur. As long as enough precursors are inside, more carbyne is formed, while carbyne is etched, i.e., broken down after most precursor has already been converted to inner nanotubes or carbyne. After the etching of some inner walls and chains, the resulting carbon inside can be used as new precursor for the formation of more carbyne, leading to an increase in yield once more. This might be the case for the 1.41 nm eDIPS nanotubes, where the highest bulk yields of around 26% and 27% were measured after two and six steps. In Figure 3b, the realized growth potential for each nanotube host type is plotted, based on the CNT hosts containing only a certain fraction of tubes with the appropriate diameter for carbyne growth. Maximum fillabilties of 48%, 31% and 35% were determined from optical absorption (see Supporting Information S1–S3) for the 1.45 nm arc‐discharge, the 1.33 nm eDIPS and for the 1.41 eDIPS hosts, respectively. For the 1.45 nm arc‐discharge peapods, with 96%, almost the full growth potential could be achieved while 90% of the growth potential was realized for the 1.33 nm sample. For the 1.41 nm eDIPS sample, 77% realized growth was accomplished.

The yield results from a complex interplay between etching and growth of inner walls and carbyne chains. Therefore, the knowledge of the realized growth potential is essential to determine the optimal number of annealing and oxidation steps. When comparing the two different eDIPS host tubes, the bulk yield is not improved by additional filling with C_60_ fullerenes. A possible explanation is the general availability of amorphous carbon inside eDIPS CNTs from their synthesis process, indicating that the limiting factor for CC growth inside eDIPS host tubes is not the carbon source. In comparison, the yield for the “cleaner” arc‐discharge host tubes was significantly improved by filling with C_60_ fullerenes, as discussed in Section 2.1.

### 2.4 Re‐Evaluating the Optimal Synthesis Temperature

**Figure 4:**
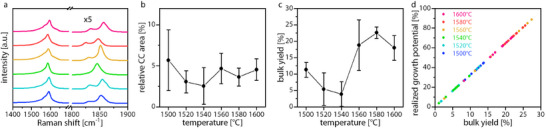
Temperature optimization for CC synthesis: a) Raman spectra of CC synthesized from 1.33 nm eDIPS nanotube hosts at different temperatures from 1500°C (bottom) to 1600°C (top) in steps of 20°C, measured using a 568 nm laser. The mode of the CC at around 1850 cm^−1^ was magnified by a factor of five; b) relative area of the LO‐peak of CC at different temperatures between 1500 and 1600°C from multiple spots on each sample; c) bulk yield calculated using the coupled mode evaluation model from the same experiment; d) bulk yield and realized growth potential calculated for all spectra.

In previous studies, temperatures of 1500°C or slightly below were used for high temperature annealing.^[^ ^26^, ^29^, ^30^, ^31^, ^35^ ^]^ Since it was shown in Section 2.3 that the full growth potential was not yet realized for the eDIPS host tubes, it was investigated if higher annealing temperatures could improve the yield. Additionally, the novel method for yield determination (coupled mode evaluation model) was compared to the established practice of comparing the areas of the CC‐peaks at around 1850 cm^−1^.^[^ ^29^ ^]^ For this experiment, eDIPS CNTs with a mean diameter of 1.33 nm were used as host tubes. The nanotube samples were annealed in HV for 1 h with annealing temperatures between 1500 and 1600°C in steps of 20°C. The eDIPS nanotubes were shown to have enough carbon inside from the synthesis process so carbyne can grow without filling with a carbonaceous precursor. The associated Raman spectra are shown in **Figure** **4**. For each sample, at least 10 spots were measured to determine the average bulk yield (see Supporting Information S4).

From the analysis of the CC‐mode alone (Figure 4b) one might conclude that higher temperatures do not significantly improve the CC yield. However, the relative CC‐peak area is not directly related to the bulk yield. The CC‐mode intensity strongly varies among different spots, as the signal is dependent on the amount of carbyne chains in resonance with the specific laser. Therefore, using our newly developed model which is independent of the resonance behavior of CC, we could determine the actual trend of the bulk yield (Figure 4c). Our results show that the optimal temperature for the synthesis of CC inside 1.33 nm eDIPS hosts is around 1580°C, with an average bulk yield of 23% and an average realized growth potential of 73% (Figure 4d).

## 3 Conclusion

Several parameters for the synthesis of carbyne confined inside different CNT hosts and their influence on the bulk yield were quantitatively evaluated utilizing a novel method of yield determination.^[^ ^38^ ^]^ Filling the CNT hosts with a carbonaceous precursor strongly improves the yield for arc‐discharge CNT hosts. Repeating the annealing step with an oxidation step in‐between also increases the bulk yield. Whether only DWCNTs are grown in the first step (1300°C) or there is inner wall and carbyne growth (1500°C) at the same time was not found relevant. When testing a multi‐step procedure with more repetitions of high temperature annealing, the highest yields were observed after four steps for some samples, with additional steps leading to lowering of the yield. For another sample, the highest yields were observed after two and six steps, with lower yields observed for the steps in‐between. Therefore, we propose a mechanism of concomitant carbyne growth from carbon feedstock and destruction of the carbyne due to residual gas inside the furnace. The availability of carbon feedstock limits the growth and the interplay of the two mechanisms determines whether the yield increases or decreases. Using the new model for yield quantification, it was determined that the optimal temperature for high temperature HV annealing for the eDIPS host tubes is around 1580°C. These findings show that the optimal synthesis temperature and number of annealing steps may vary when using different host nanotubes. For comparability, here, the same oxidation and annealing experimental conditions were applied to all three of the samples. However, each sample has different optimal conditions depending on the carbonaceous material inside and the diameter distribution. During the oxidation process, the stability of the CC@DWCNTs strongly depends on the diameter/chirality of the inner and outer tubes, which is dependent on the synthesis and separation methods used to prepare the SWCNT hosts. This underlines the importance of reliable methods for yield determination and the usefulness of the realized growth potential as a metric for the success of the synthesis and allows optimization of the synthesis pathway for individual samples. Here, optimizing the relative fraction of the carbonaceous precursors by stepwise re‐oxidation and annealing is a suitable pathway to achieve the growth of CC@DWCNT with close to 100% growth efficiency.

## 4 Experimental Section

The CC@DWCNT samples were prepared using a ceramic furnace set‐up with ultra‐high vacuum at room temperature and high vacuum (<10^−5^ mbar) at the annealing temperatures. The details of the synthesis are discussed in the relevant sections in the results chapter. The yield was determined using the coupled mode evaluation model, based on the changes in the G‐mode of CC@DWCNT samples compared to unfilled DWCNTs.^[^ ^38^ ^]^ The area of the G‐mode of the DWCNTs is subtracted from the G‐line area of the CC@DWCNT sample and then divided by the G‐line area of the CC@DWCNT sample in order to obtain the relative area percentage of the G‐mode contributed by the carbyne chain. This was then calibrated to obtain absolute bulk yield values. The realized growth potential referenced in the results section refers to the yield related to the amount of CNT hosts with a suitable diameter for carbyne growth. The diameter distribution of all samples were determined using optical absorption spectroscopy (see Supporting Information S1–S3). The optical absorption measurements were made with a Bruker vertex 80v spectrometer. The measurements were performed on thin films on BaF_2_ wedges.

The challenges of systematic investigations into carbyne synthesis parameters are the inhomogeneity of the samples and the lack of exact control over some of the parameters, such as the pressure inside the furnace during the high temperature HV annealing. The issue of inhomogeneity was mitigated by measuring multiple spots depending on the size of the sample in order to obtain robust averages. For example, for the temperature evaluation, at least 10 spots were measured on each sample (see Supporting Information). For the evaluation of the optimal number of annealing steps, the same spot on the sample was measured after each step to ensure comparability. Additionally, samples can be put into the furnace together to ensure the same external parameters, such as temperature and pressure, for optimal comparability.

The Raman measurements of all samples were made with a Horiba LabRAM HR spectrometer combined with an Ar^+^/Kr^+^ laser (Coherent Innova 70C). All spectra were measured with a wavelength of 568 nm. As detector, a liquid nitrogen cooled CCD chip was used.

All structure models were made using VESTA.^[^ ^42^ ^]^

### Statistical Analysis

The Raman spectra were normalized to the intensity of the G‐line. Average values from different spots on the samples (Figure 2) correspond to the mean ± the standard deviation. In Figure 4b,c, the sample sizes were 10 to 12 (see Supporting Information S4). Mean values are plotted, the standard deviation is shown as error bars. For the area estimations in the optical absorption spectra (Supporting Information S1–S3), a linear background subtraction was performed.^[^ ^43^ ^]^

## Conflict of Interest

The authors declare no conflict of interest.

## Supporting information

Supporting Information

## Data Availability

The data that support the findings of this study are available from the corresponding author upon reasonable request.
