# Supplementary material for: Systematic Optimization of the Synthesis of Confined Carbyne
Source: Small Methods. 2025 Apr 9;9(8):2500075. doi: 10.1002/smtd.202500075 (PMC12391636; doi:10.1002/smtd.202500075)
Supplement: Supplementary file 1 — Supporting Information [file SMTD-9-2500075-s001.pdf]

small methods

Supporting Information

for *Small Methods*, DOI 10.1002/smtd.202500075

Systematic Optimization of the Synthesis of Confined Carbyne

Clara Freytag, Christin Schuster, Emil Parth, Dido Denier van der Gon, Takeshi Saito, Kazuhiro Yanagi, Paola Ayala and Thomas Pichler**

Systematic Optimization of the synthesis of confined carbyne: Supporting information

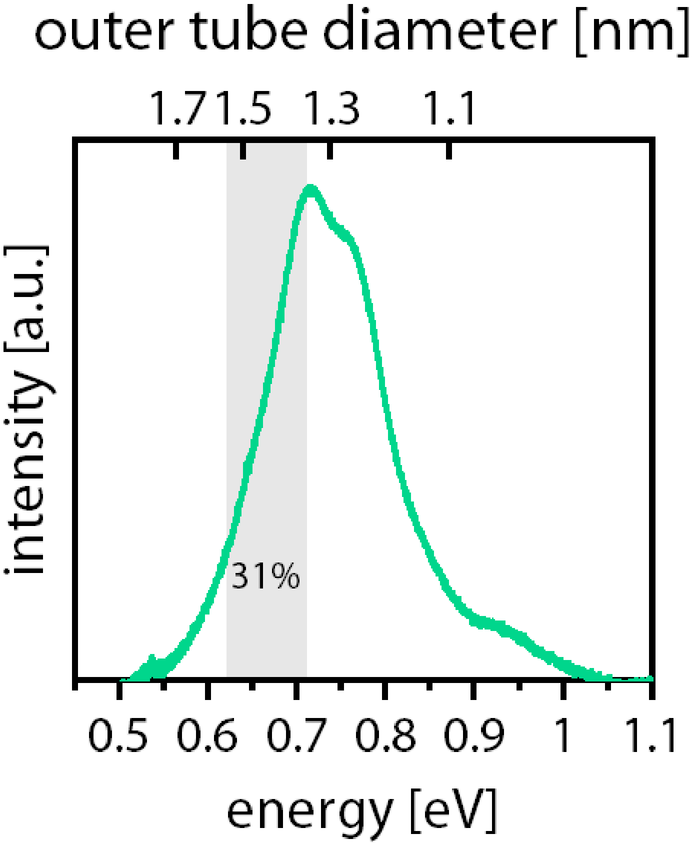

Figure S1: Assumed diameter distribution for the 1.33 nm eDIPS host tubes. The gray box indicates the region in which the resulting inner tubes (diameter of the outer tubes -0.72 nm [1]) are expected to grow chains [2]. The integration was done between the boundaries of 1.35 nm and 1.55 nm. Around 31% of the material can theoretically be filled with CC. The conversion of energy to diameter was done according to Liu *et al.* [3].

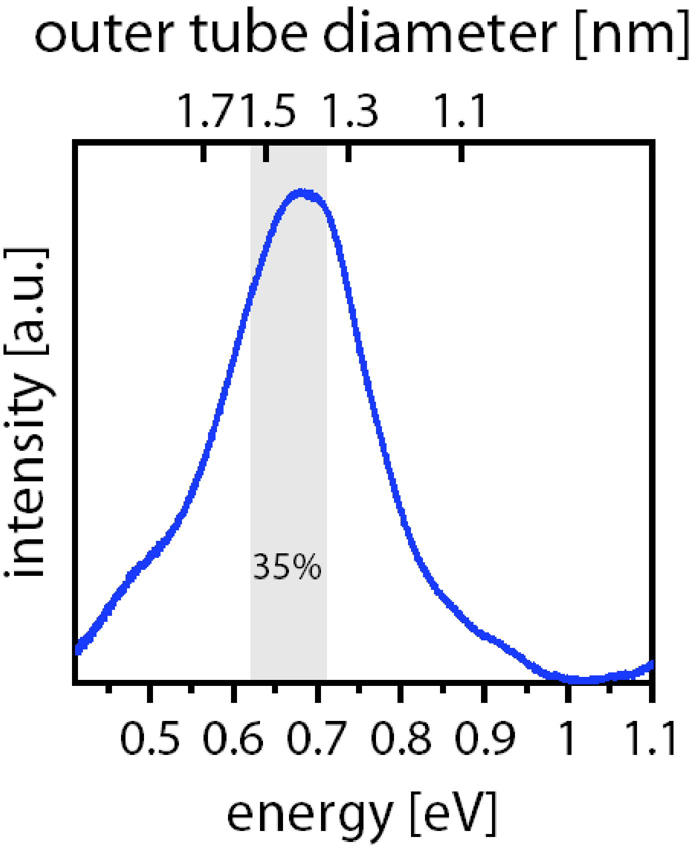

Figure S2: Assumed diameter distribution for the 1.41 nm eDIPS host tubes. The gray box indicates the region in which the resulting inner tubes (diameter of the outer tubes -0.72 nm [1]) are expected to grow chains [2]. The integration was done between the boundaries of 1.35 nm and 1.55 nm. Around 35% of the material can theoretically be filled with CC. The conversion of energy to diameter was done according to Liu *et al.* [3].

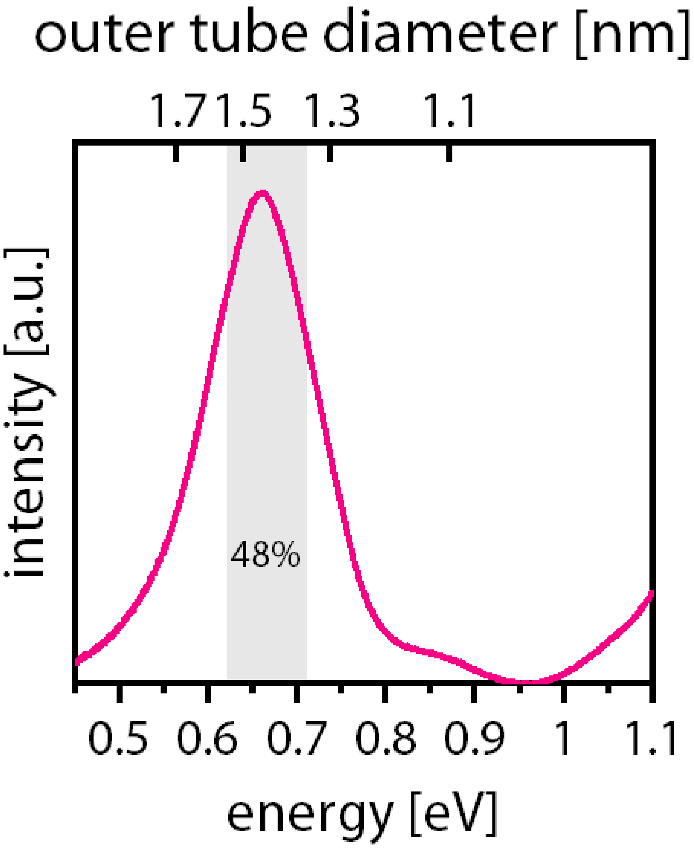

Figure S3: Assumed diameter distribution for the 1.45 nm arc-discharge host tubes. The gray box indicates the region in which the resulting inner tubes (diameter of the outer tubes -0.72 nm [1]) are expected to grow chains [2]. The integration was done between the boundaries of 1.35 nm and 1.55 nm. Around 48% of the material can theoretically be filled with CC. The conversion of energy to diameter was done according to Liu *et al.* [3].

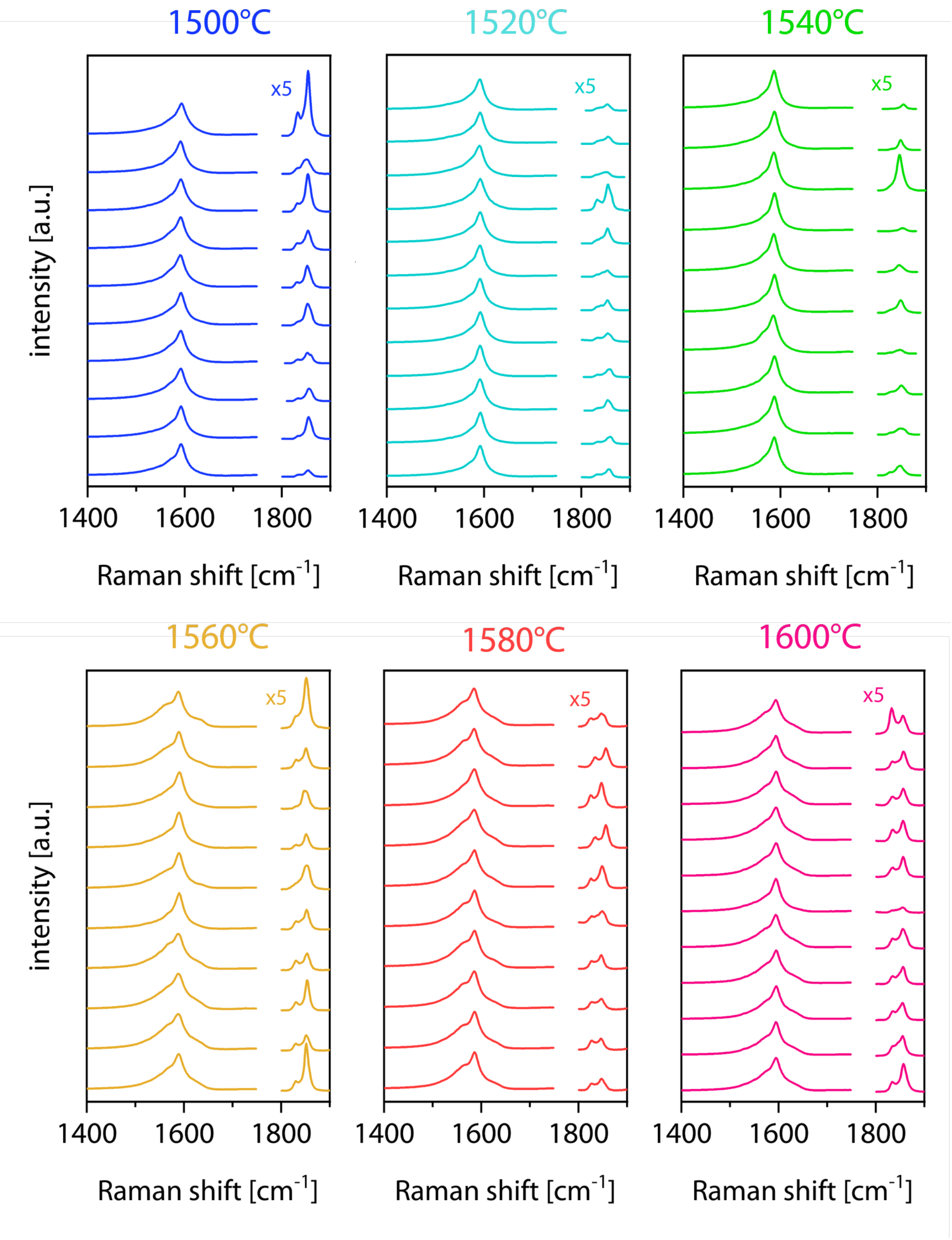

Figure S4: Raman spectra of confined carbyne synthesized from 1.33 nm eDIPS nanotube hosts at different temperatures from 1500°C to 1600°C in steps of 20°C, measured using a 568 nm laser. At least 10 spots were measured on each sample in order to obtain averages and standard deviations shown in the main text in figure 4b and c.

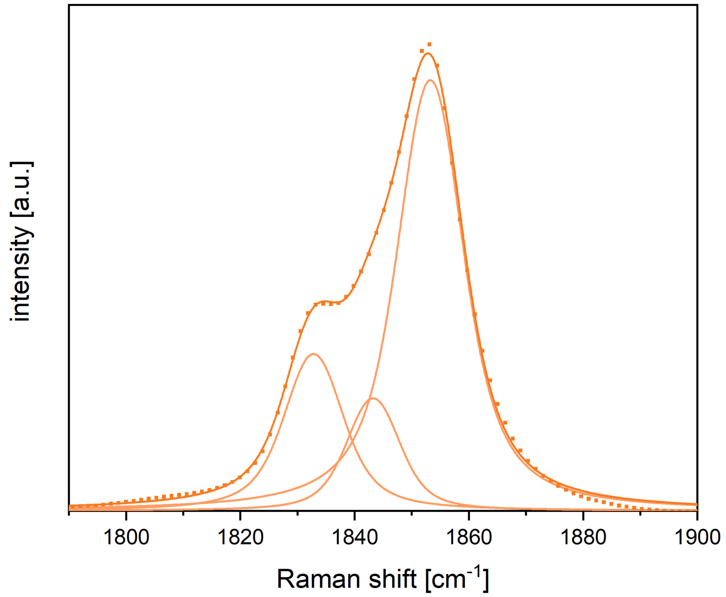

Figure S5: Line-shape analysis of the CC-mode for the CC@DWCNT sample grown from peapods (Fig.1 in the main text)

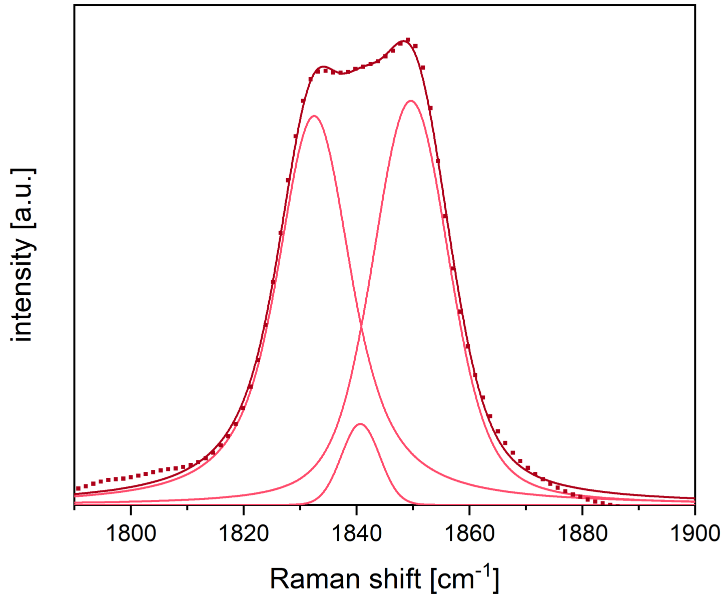

Figure S6: Line-shape analysis of the CC-mode for the CC@DWCNT sample grown from unfilled SWCNTs (Fig.1 in the main text)

References

[1] M. Abe, H. Kataura, H. Kira, T. Kodama, S. Suzuki, Y. Achiba, K.-i. Kato, M. Takata, A. Fujiwara, K. Matsuda, Y. Maniwa, *Physical Review B* **2003**, *68*, 4 041405.

[2] S. Heeg, L. Shi, L. V. Poulikakos, T. Pichler, L. Novotny, *Nano Letters* **2018**, *18*, 9 5426.

[3] X. Liu, T. Pichler, M. Knupfer, M. S. Golden, J. Fink, H. Kataura, Y. Achiba, *Phys. Rev. B* **2002**, *66* 045411.
